# Lactylation Modification and Aging: A Molecular Link in the Life Process

**DOI:** 10.14336/AD.2025.0509

**Published:** 2026-07-27

**Authors:** Min Shi, Honyu Li, Haiyan Lin, Qiang Tang

**Affiliations:** Heilongjiang University of Chinese Medicine, Harbin, Heilongjiang 150040, China

**Keywords:** Lactylation modification, Aging, Aging mechanisms

## Abstract

In recent years, significant progress has been made in the research of aging and aging-related diseases. Epigenetic alterations, as an important marker of aging, are also one of the mechanisms that trigger aging. Lactylation modification, also known as lactylation, is a post-translational modification form (PTM) that uses lactate and lysine residues as substrates and belongs to an important field of epigenetic alterations. As an emerging research hotspot in recent years, it has made considerable breakthroughs in the study of human-related diseases, especially cancer. During the aging process, lactylation modification occurs in the body due to changes in lactate concentration, thereby affecting other aging mechanisms. This article reviews lactylation modification and cellular senescence, mitochondrial dysfunction, autophagy and energy metabolism, summarizes the current influence of lactylation modification on aging-related mechanisms, and makes bold predictions for future research based on the existing research differences.

## 1. Introduction

Aging is a series of irreversible physiological function decline processes that occur over time after the normal growth and maturity of the organism. It is characterized by a progressive deterioration of physiological integrity, leading to impaired functional capacity and, ultimately, increased susceptibility [1]. Modern medicine has always regarded anti-aging research as a core research field. By exploring the mechanism of aging and innovating diagnosis and treatment technologies, it continues to improve the prevention and clinical intervention level of aging-related diseases, hoping to effectively alleviate the pressure on medical resources and economic burden caused by the aging society, and promote medical theoretical breakthroughs and technological innovations. However, a recent authoritative study pointed out that although many advances have been made in modern biological gerontology, there are still seven significant knowledge gaps at the basic theoretical level. These seven knowledge gaps are further classified into three categories. Namely, the evolutionary biology mechanism of longevity traits, the regulatory network of survival and death of organisms, and the formation of phenotypic heterogeneity among individuals during aging [2]. Among them, aging phenotypes are highly heterogeneous at all levels of biological tissues. This gap is particularly prominent at the molecular level. Unwinding, quantifying, explaining, and recognizing the causes and consequences of phenotypic heterogeneity in aging were also identified as the most challenging knowledge gaps. Based on the research in 2013, the Carlos Lopes-Otin team proposed twelve major signs of aging, among which epigenetic changes are a key one, mainly covering abnormal DNA methylation patterns, disordered post-translational modifications of histones, dysregulation of chromatin remodeling and dysfunction of non-coding RNA [3].

**Figure 1.**
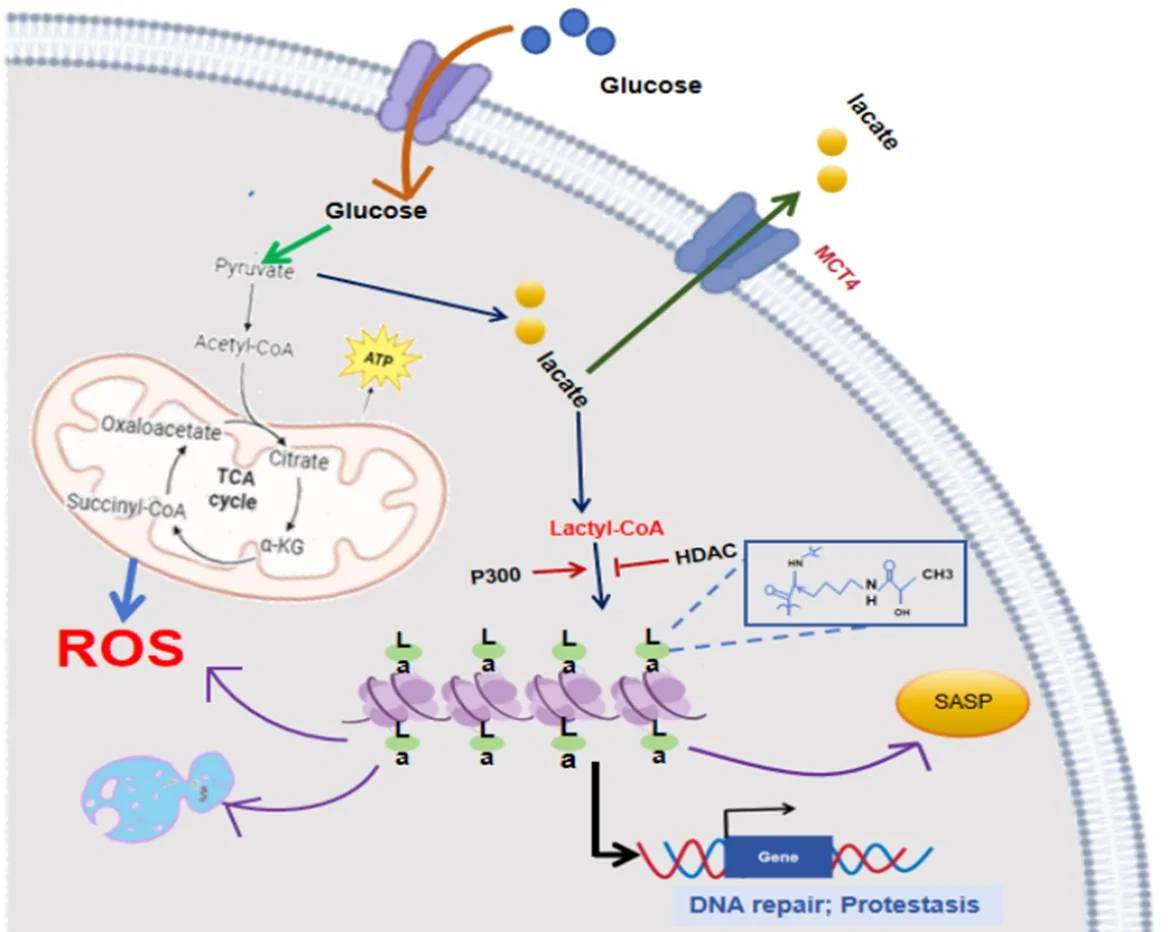
**During the aging process, lactate produced by glycolysis, under the catalysis of specific enzymes, binds to specific protein residues, thereby undergoing lactylation modification**. This modification can influence the gene expression patterns in the body. In addition, by influencing mitochondrial oxidative stress and autophagy, it mediates the release of SASP, leading to the occurrence of cellular senescence, thereby promoting the aging process and facilitating the occurrence of aging-related diseases.

A plethora of post-translational modifications (PTMs) exist for proteins within the human body. These modifications are categorized under the realm of epigenetics, as they do not directly alter the DNA sequence itself. Instead, they exert their influence on gene expression and modulate the functional activity at the protein level [4]. As mass spectrometry technology continues to advance, an ever-growing array of PTMs has been uncovered. These modifications can be neatly classified based on the covalent attachment of distinct small molecules to amino acid residues. The categories include phosphorylation, glycosylation, SUMOylation, ubiquitination, acetylation, and methylation [5, 6]. Lactylation modification, also known as lactylation, is an emerging form of post-translational protein modification. It is a PTM based on lactic acid and lysine residues. As an epigenetic regulatory factor, it can regulate intracellular signal transduction, gene expression and protein function [7]. Under normal physiological conditions, lactate is primarily produced in anaerobic conditions. The liver plays a key role in its removal through two main pathways: gluconeogenesis, which is part of the Cori cycle, and ATP production via oxidative phosphorylation and the Krebs cycle [8]. In 2019, a groundbreaking study revealed that the accumulation of lactate can induce histone lactylation, thereby exerting a significant influence on gene transcription. Moreover, this study meticulously identified and mapped a total of 28 lactylation sites on core histones in both human and mouse cells, shedding new light on the intricate mechanisms underlying lactate's role in epigenetic regulation [9]. As age increases, the level of lactate in the body rises correspondingly. This elevation in lactate levels leads to varying degrees of lactylation modification. By modulating aging-related mechanisms, lactylation modification exerts a significant impact on the aging process and the development of aging-related diseases [10] (Fig. 1). In the realm of aging research, the correlation between lactylation modification and aging has garnered substantial attention and emerged as a hot topic. It holds great potential for future investigations.

## 2. Principle and mechanism of lactylation modification

### 2.1 The production of lactate and the occurrence of lactylation

In normal cellular physiology, glycolysis represents a pivotal metabolic pathway for cellular energy acquisition. This process involves the stepwise conversion of glucose into pyruvic acid (pyruvate). Under aerobic conditions, pyruvate is shuttled into mitochondria where it undergoes oxidative phosphorylation (OXPHOS), yielding carbon dioxide and adenosine triphosphate (ATP) as the final products [11].Under anaerobic conditions, NADH and pyruvate generated via glycolysis undergo conversion to lactate, which is subsequently excreted from the cell. This metabolic pathway enables the production of two molecules of ATP and two molecules of lactate per glucose molecule, operating independently of oxygen availability [12]. Lactate, a key by-product of glycolysis, has traditionally been viewed as a detrimental metabolic waste under hypoxic conditions [13]. However, in chronic anaerobic or pathological states, to address the urgent energy demands, the glycolytic pathway becomes upregulated, consequently leading to excessive lactate production and intracellular accumulation. Intracellular lactate accumulation triggers enzymatic or non-enzymatic reactions of lactylation modification, forming the upstream of the metabolism-epigenetic cycle of the "metabolite (lactate) - epigenetic modification (lactylation)" axis, which links glycolytic fluences with gene expression regulation.Lactylation modifications on lysine residues include three isomers with similar structures but distinct stereochemistry: L-lactylation (KL-La), D-lactylation (KD-La), and N-ε-(carboxyethyl)-lysine (Kce). Current studies on these isomers, which focus on lysine residues, have identified their unique formation mechanisms, tissue/cell distribution patterns, and functional roles (**Error! Reference source not found.**).

**Table 1:** The differences in lactylation modification among the three isomers.

Isomers	Formation mechanism	Distribution and function	Ref.
**KL-La**	The lactylation modification is enzymatically catalyzed by acyltransferases (e.g., p300) using lactyl-CoA as a cofactor	This modification is predominantly observed in histones (e.g., H3K18la), where it regulates gene transcription by integrating metabolic and signaling pathways to facilitate cellular adaptation.	[9, 14-16]
**KD-La**	Non-enzymatic lactylation occurs through non-catalytic reactions between proteins and S-D-lactoyl glutathione (LGSH), leading to the formation of lactylated protein adducts	This non-enzymatic lactylation is predominantly enriched in glycolytic enzymes (e.g., PFKM) rather than nuclear proteins. It promotes glycolysis by allosterically modulating enzyme activity, concurrently inhibiting the Sirtuins pathway to influence anti-aging processes. Additionally, it drives the Warburg effect, reduces the NAD^+^/NADH ratio, and regulates inflammatory phenotypes.	[14, 16-18]
**Kce**	Methylglyoxal (MGO), a glycolytic by-product, undergoes non-enzymatic reactions with lysine residues, leading to protein modification	MGO predominantly localizes to non-histone proteins while exhibiting negligible levels in histones. It promotes glycolytic gene expression by stabilizing HIF-1α, thereby establishing a pro-metabolic positive feedback loop	[19-21]

Lactylation is primarily driven by lactate generated during metabolic processes, with endogenous lactate produced via glycolysis serving as a critical determinant of lactate levels [22]. Moreover, exogenous lactate (e.g., exercise-induced lactate) also exerts a modulatory effect on body lactate homeostasis. Most crucially, lactate from these two distinct sources (endogenous vs. exogenous) exhibits opposing roles in the aging mechanism.

Endogenous lactate production promotes aging progression by two major mechanisms. Firstly, it induces chronic inflammation and cellular senescence via histone lactylation, thereby disrupting epigenetic regulation [23]. In microglia of Parkinson's disease (PD) mice, a significant upregulation of H3K9 lactylation is observed. Inhibition of glycolysis with 2-deoxy-D-glucose (2-DG) attenuates neuroinflammation-induced microglial proliferation and neuronal injury by reducing lactate accumulation [24].

Compared with endogenous lactate, exogenous lactate exerts an opposing effect on the aging mechanism. Lactate produced via exercise demonstrates neuroprotective effects by markedly enhancing the lactylation of various synaptic proteins. This, in turn, improves synaptic function and modulates the phenotypic transformation of microglia, thereby alleviating neuroinflammation. Long-term high-intensity interval training (HIIT) and lactate administration have positive impacts on brain function in aged mice [25-27]. In the context of aging-related diseases associated with osteoporosis, exercise can counteract the reduction in lactate levels caused by the specific loss of endothelial cell pyruvate kinase M2 (Pkm2) within endothelial cells. This process mediates histone lactate-dependent differentiation of bone marrow stromal cells (BMSC) into osteoblasts, thereby promoting bone formation and indirectly improving the incidence of osteoporosis [28]. The L-lactate dehydrogenase B chain (LDHB) is emerging as a promising therapeutic target for osteoporosis. Serving as a downstream effector of the osteoblast signal transduction factor and immediate early response 3 (IER3), LDHB holds a critical position in bone metabolism. Notably, inhibition of IER3 expression blocks the exercise-induced mechanical stress-mediated increase in lactate content and lactylation levels in bone marrow stromal cells (BMSCs) [29]. In aging skeletal muscle, dysregulation of the enzyme leads to the loss of histone lactylation in the skeletal muscle, disrupts the steady state of protein and DNA repair, and accelerates muscle aging. Running increases exogenous lactate levels, enhances histone lactylation, and thereby slows down the aging rate of skeletal muscles [30].

### 2.2 Regulatory mechanisms of lactylation

Lactate plays a crucial role in many cellular processes by regulating gene transcription through lactoylation of histone and non-histone proteins. These processes include inflammation, embryonic development, tumor proliferation, and metabolism [31]. Recent proteomic studies have documented lactoacylation modifications in a wide spectrum of proteins across nuclear, cytoplasmic, and mitochondrial subcellular niches [32]. During lactylation, lactosyltransferases mediate the catalytic attachment of lactoyl moieties, thereby orchestrating dynamic regulation of this post-translational modification.

The common lysine lactyltransferase is p300, which predominantly mediates histone lysine lactylation modification. Its overexpression or depletion can respectively increase or decrease the cellular level of histone Kla [33]. In addition, AARS1 functions as a lactoyltransferase by recognizing and catalyzing lactate activation, forming an activated intermediate that subsequently enables lactate to bind to lysine residues on target proteins, thereby inducing lactylation modification [34].AARS1 also translocates to the nucleus, where it directly employs lactate as a lactoyl donor and ATP as an energy substrate to catalyze lactoacylation of Yes-associated protein (YAP) at lysine 90 and TEA domain transcription factor 1 (TEAD1) at lysine 108 [35].

On the other hand, deacylase regulates lactylation modification by removing the lactoyl group. The mechanisms can be categorized into enzymatic and non-enzymatic reversal. The enzymatic reversal mechanism is currently primarily associated with the Sirtuin family and HDACs. SIRT2 is currently a widely verified delactoylating enzyme, with delactylation activity significantly higher than Class I HDACs (HDAC1-3). It catalyzes ester bond hydrolysis by consuming the cofactor NAD^+^, and SIRT2 binds to NAD^+^ and lactates the substrate. The nicotinamide moiety of NAD^+^ is hydrolyzed to form an unstable ADP-ribose intermediate; this intermediate undergoes spontaneous hydrolysis, releasing free lactate and regenerating the lysine amino group. Importantly, during macrophage polarization, SIRT2 specifically removes lactylation modifications at pro-inflammatory gene loci (e.g., TNF-α promoter), thereby promoting M1-to-M2 transition and inhibiting senescence-associated inflammation.” [36]. SIRT6, an NAD-dependent nuclear deacylase, targets lysine residues in histones (e.g., H3K27) and removes their lactylation modifications through conformational changes. Its dysregulation impairs chromatin stability, upregulates senescence-associated genes (e.g., p16 INK4a), and accelerates aging and age-related diseases. [37]. SIRT3, a mitochondrial NAD-dependent deacylase, erases lactylation of histone H3K9 and cyclin E2 (CCNE2). By activating mitochondrial oxidative phosphorylation (OXPHOS) genes (e.g., mtCO1), it maintains energy metabolism homeostasis, thereby suppressing cellular senescence and aging-related pathologiesr [38, 39].

In *in vitro* experiments, Class I histone deacetylases (HDAC1-3) exhibited relatively weak delactylation activity. They rely on zinc ions at the active site to activate water molecules and directly hydrolyze Kla ester bonds. However, their efficiency is significantly lower than that of SIRT2. The physiological significance of this activity still needs to be further verified through *in vivo* experiments [40, 41]. A study, through a systematic analysis of the acyl specificity of human zinc-dependent HDAC and sirtuin subtypes, found that HDAC3, as a robust delactase, has an activity thousands of times higher than that of SIRT2 [42].HDACs 1 and 3 can significantly alter the acylation level of the H4K5 site. HDAC1 and HDAC2 are often found in the same multi-protein complex in the nucleus and are considered to compensate for each other in the regulation of histone Kac [43]. Meanwhile, HDAC1-3 display potent deacylase activities toward various modifications, including K(L-la), K(D-la), and diverse short-chain acyl modifications [44].

Non-enzymatic reversal is primarily attributed to acidic pH-induced hydrolysis. When the local microenvironment pH decreases, the carbonyl group of the lactoyl ester bond undergoes protonation, thereby enhancing its electrophilicity. Subsequently, a water molecule nucleophilically attacks the carbonyl carbon, breaking the ester bond and releasing free lactate [45, 46]. The accumulation of lactate acidifies the microenvironment, thereby accelerating non-enzymatic hydrolysis and forming a metabolism-epigenetic negative feedback loop [47]. A non-enzymatic acylation of Lys derived from LGSH is fundamentally present and abundant in highly glycolytic tissues that are rich in glycolytic enzymes. This modification, LactoylLys, leads to a decrease in glycolytic output by inhibiting enzyme activity and reducing glycolytic metabolism [48]. Considering the acidic pH of brain tissue in neuropsychiatric disorders results from mitochondrial impairment and lactate accumulation, histone lactoacylation can facilitate chromatin accessibility via a mechanism dependent on oxidative energy metabolism [49].

## 3. Physiological function of lactylation modification

### 3.1 Regulates gene expression

Histone lactylation, such as H3K18la, is an emerging epigenetic modification. It plays a central role in the regulation of gene expression by dynamically altering chromatin structure (such as opening or compaction) and directly affecting transcription factor binding ability [50, 51]. It is not only a biomarker of active chromatin but also couples the cellular metabolic state (especially lactate produced by glycolysis) with chromatin organization and the activation of the gene regulatory network (GRN). For example, during embryonic development, lactylation marks specific regions (such as the neural crest) and regulates key loci [52, 53]. In cell reprogramming, Glis1 promotes glycolytic upregulation, thereby elevating lactate and acetyl-CoA levels. This metabolic rewiring enhances acetylation (H3K27Ac) and lactylation (H3K18la) at pluripotency gene loci, forming a signaling cascade that drives reprogramming [54].

Lactoylation of lysine 271 in Mecp2 (Mecp2K271la) inhibits the expression of epithelial regulatory protein (Ereg) by mediating chromatin binding, thereby suppressing atherosclerosis [55]. Furthermore, histone lactoylation promotes the transcription of repair-related genes, thereby modulating the anti-inflammatory and angiogenic functions of monocyte-derived macrophages. This dual regulation establishes a permissive microenvironment for cardiac repair after myocardial infarction (MI) [56]

### 3.2 Mediate signaling

In the intricate cellular signal transduction network, lactylation modification holds a pivotal role. Upon lactylation, the catalytic activity of certain protein kinases or phosphatases involved in signal transduction can be altered, thereby influencing the efficiency and direction of signaling pathway transmission.

Chronic inflammation is a hallmark of aging, and macrophage polarization is crucially involved in inflammatory regulation. Intriguingly, emerging evidence shows that histone lactylation modulates the M1/M2 phenotypic transition of macrophages [57]. Transcriptomic analysis indicated that H3K9 lacto-acylation regulates M1 macrophage polarization and inflammatory responses through the tumor necrosis factor (TNF) signaling cascade. Pharmacological inhibition of P300 or genetic downregulation of lactate dehydrogenase A (LDHA) expression blunted H3K9 lactoacylation and suppressed M1 phenotypic polarization [58]. Upon VEGF stimulation, the level of H3K9 lactylation (H3K9la) is upregulated in endothelial cells. This modification forms a feedback loop with histone deacetylase 2 (HDAC2) to drive VEGF-induced angiogenesis [59]. In addition, there is a feedback loop between H4K12 lactacylation (H4K12la) and histone deacetylase 3 (HDAC3) in macrophages, which drives lactacide-induced collagen synthesis. In this way, it can reduce the loss of collagen from fibroblasts and inhibit skin aging [60]. Furthermore, in a study, an epigenetic modification of the LDHA-NDRG1 axis driven by lactate was proposed to resist the occurrence of aging [61].

### 3.3 Regulation of energy metabolism

Lactylation modification is of great significance in the regulation of cellular metabolism and can directly regulate the activities of key enzymes in metabolic pathways. Lactylation modulates the activity of metabolic enzymes and transcription of metabolic genes, forming the ‘epigenetic modification (lactylation) - metabolic phenotype’ feedback—the downstream of the cycle, which reshapes cellular metabolic status [62]. IIn glucose metabolism, the activities of key enzymes such as phosphofructokinase change after lactylation modification, affecting the flow and direction of the metabolic pathway. Studies indicate that lactoyl-modified proteins in the prokaryote Escherichia coli primarily participate in metabolic regulation. For example, CobB modulates PykF activity by removing lactoyl groups (Kla) from lysine 382 (K382), thereby promoting glycolysis and bacterial proliferation [63]. Moreover, the lactylation level is positively correlated with the degree of glycolysis reprogramming and can regulate glycolysis by affecting enzyme transcription and structural functions [64].

In lipid metabolism, lactylation modulates the activity of lipogenic and lipolytic enzymes, thereby contributing to lipid accumulation or metabolic dysregulation. For instance, studies have demonstrated that lactate and its receptor GPR81 inhibit lipolysis by decreasing intracellular cAMP levels in adipocytes [65]. Upon inhibition of blood lactate elevation, the fat-reducing effect of HIIT (high-intensity interval training) was attenuated, demonstrating that lactate is critical for HIIT-mediated lipolysis. These findings suggest that lactylation, as a negative feedback regulatory mechanism, serves as a key link between glucose and lipid metabolism [66].

In this way, lactylation modification can coordinate the balance between different metabolic pathways in the cell and ensure that the cell can maintain normal metabolic function under different nutrient and energy states.

## 4. The interaction between lactylation modification and other post-translational modifications

### 4.1 Acetylation

As a critical post-translational modification (PTM), acetylation exerts profound effects on the functions of various proteins, such as histones, p53, and tubulin [67]. Major forms of acetylation comprise N-terminal acetylation, histone acetylation, and C-terminal residue acetylation [68]. Significantly, N-terminal acetylation is irreversible, and lacks identified deacetylases, thus exerting a sustained influence on protein stability, subcellular localization, and intermolecular interactions. This irreversibility carries particular significance in aging-associated disorders [69, 70]. While the irreversibility of N-terminal acetylation is well-established, mechanistic insights into how it specifically orchestrates protein functions, influences pathological cascades, and whether non-enzymatic or indirect regulatory mechanisms sustain its persistence in aging and disease remain limited.

Acetylation and lactylation together constitute the central hub of cellular metabolic status and epigenetic regulation. Investigative evidence has demonstrated that Glis1 directly engages with glycolytic gene promoters to promote chromatin accessibility while concurrently repressing chromatin at somatic gene loci, thereby orchestrating glycolytic regulatory networks. Consequentially, heightened glycolytic flux elevates intracellular acetyl-CoA and lactate concentrations, thereby augmenting acetylation (H3K27Ac) and lactoacylation (H3K18la) at pluripotent gene loci to facilitate cellular reprogramming [54].Acetylation can directly regulate the lactylation level. Component X of the pyruvate dehydrogenase complex (PDHX) is acetylated by p300, which disrupts the assembly of the pyruvate dehydrogenase complex (PDC). This disruption redirects pyruvate metabolism toward lactate production, thereby promoting H3K56 lactylation and oncogene expression [71]. On the other hand, histone lactylation compets with acetylation for epigenetic modifications of lysine and marks the levels of lactate and acetyl-CoA. The metabolic bifurcation of pyruvate into lactate or acetyl-CoA during glycolysis dictates the cellular trajectory toward malignant transformation [72]. This competitive relationship is particularly evident in PKM2. P300 acts as both an acetyltransferase (acetylating PKM2 at K433) and a lactoyltransferase (promoting PKM2 lactylation), and these two modifications mutually inhibit each other. Notably, mannose reduces PKM2 lactylation while enhancing its acetylation; conversely, lactate supplementation increases PKM2 lactylation and suppresses its acetylation [73].

### 4.2 Ubiquitylation

Ubiquitination is a PTM in eukaryotic cells. Through the E1 (activating enzyme)-E2 (conjugating enzyme)-E3 (ligase) enzymatic cascade, ubiquitin is covalently attached to substrate proteins. Ubiquitinated proteins are thereby labeled for proteasomal degradation [74-76].

Lactylation exerts bidirectional regulation on ubiquitination: on one hand, it inhibits ubiquitin-mediated proteasomal degradation of substrate proteins by disrupting their interaction with E3 ligases. For example, lactate molecules covalently lactoyl-modify the transcription factor EB (TFEB), promoting its lactylation and stabilization. This modification prevents TFEB from binding to the E3 ubiquitin ligase WWP2, thereby inhibiting TFEB ubiquitination and proteasomal degradation, which in turn enhances autophagic flux [77].Intracellular LPS detection by Caspase-11 mediates non-classical pyroptosis. In acetaminophen-induced acute liver injury, lactate elevates Caspase-11 levels. Mechanistic studies show lactate promotes NEDD4 K33 lactylation, inhibiting its interaction with Caspase-11 (negative regulator) and suppressing Caspase-11 ubiquitination [78]. Lactate promotes cGAS lactylation, inhibiting binding to E3 ligase MARCHF5, blocking cGAS degradation, and driving robust IFN-I response in SLE patients [79]. IGFBP7 is associated with α-enolase (ENO1) to suppress its ubiquitination and degradation. Elevated ENO1 promotes glucose metabolic reprogramming and lactate accumulation. Conversely, lactate augments IGFBP7 transcription via histone H3K18 lactoacylation. Significantly, IGFBP7-targeted therapy ameliorates cadmium-induced hepatic and renal fibrosis [80].

On the other hand, lactate can also promote the ubiquitination degradation of specific proteins or enhance the expression of E3 ligases. Hypoxia treatment induces lactylation of Axin1 protein and promotes the ubiquitination of Axin1 for protein degradation, thereby providing its antiglycolytic function [81]. Hyper-glycemia-induced lactate promotes AARS1-mediated YTHDC1 lactylation and enhances its ubiquitination. This study identifies a hyperglycemia-lactate-AARS1-YTHDC1-Jund-NECtin4 axis regulating breast cancer (BC) extracellular vesicle (EV) sensitivity [82]. Histone H3K18 lactylation promotes HECTD2 transcription. As an E3 ubiquitin ligase for KEAP1, HECTD2 facilitates KEAP1 degradation [83]. Active glycolysis promotes heightened lactoacylation, primarily histone lactoacylation, with H3K14la being pivotal for gene regulation. NEDD4, a ubiquitin E3 ligase, acts as a downstream target of H3K14la. Histone lactoacylation-regulated NEDD4 interacts with PTEN to mediate its ubiquitination and degradation [84].

## 5. Changes in lactate levels during aging

Lactate, a major glycolytic by-product, is primarily generated from pyruvate via lactate dehydrogenase (LDH) [85]. While traditionally viewed as a metabolic waste, lactate exhibits abnormal glycolytic characteristics in tumor cells—excessive production even under aerobic conditions. First described by Otto Warburg, this phenomenon is known as the "Warburg effect" [86]. Studies show lactate levels rise with aging, mechanistically linked to mitochondrial dysfunction. Aging-induced deterioration of mitochondrial structure and respiratory chain efficiency impairs cellular oxygen utilization. To maintain energy homeostasis, cells shift to glycolysis, generating pyruvate that converts to lactate under hypoxic-like conditions, driving intracellular lactate accumulation—particularly evident in the brain [87].

A study analyzed mitochondrial function and respiratory chain enzymes in key brain regions of normal and progeria mice. In situ hybridization showed brain lactate elevation resulted from altered lactate dehydrogenase transcriptional activity, promoting pyruvate-to-lactate conversion [88].The lactate dehydrogenase (LDH) inhibitor oxalate can reduce the respiration of isolated mitochondria incubated in lactate [89]. Exogenous glutamic acid treatment impairs astrocyte mitochondrial respiratory capacity, enhances aerobic glycolysis, and promotes lactate release [90].

In animal models, the brain lactate levels of normal and prematurely aged mice tripled during the aging process [88]. Lactate levels vary across brain regions, with the substantia nigra of aged mice showing the greatest increase. Mechanistic studies indicate this results from region-specific LDH-A downregulation affecting its expression and activity [91]. However, in skeletal muscle, aging-induced mitochondrial dysfunction impairs oxidative phosphorylation, reducing lactate levels and lactylation. Contrasting trends in different tissues may reflect tissue-specific aging responses [90].

**Table 2:** Quantitative summary of changes in lactate levels in some tissues during the aging process.

Tissue/Sample type	Model/ Population	lactate changes (Aging vs Youth)	Remarks	Ref.
**brain**	Mouse	Increase by 2 times (normal & premature aging	It is related to abnormal mitochondrial respiratory chain enzyme activity and changes in LDH transcriptional activity	[87, 88, 91]
**np cell**	Rat	It increased by approximately 1.5 times (3 months old vs. 22 months old)	It is related to the inhibition of glycolysis induced by glutamine	[95]
**CSF**	Human	Increase by approximately 1.5 times (≥65 years old vs. 30-45 years old)	It is moderately positively correlated with age; It shows an upward trend after the age of 54	[92, 94]
**B cell**	Human	Anaerobic glycolytic uptake increased, but there was no specific value (≥65 years old vs. 30-45 years old)	Aging stimulates B cells to take up more glucose, and anaerobic glycolysis increases	[93]
**Skeletal muscle, Liver, Heart, Kidney**	Mouse	Decline, but no specific figure (Three months old VS 20 months old)	It is related to the decline in mitochondrial oxidation capacity and the loss of histone lactylation; Exogenous lactate (such as exercise) can reverse this trend	[30, 96]

In human studies, cerebrospinal fluid (CSF) lactate levels are higher in patients with neurodegenerative diseases over 65 years of age than in younger patients, and there is a moderate positive correlation [92]. Comparing lactate levels in 30-45-year-olds vs. ≥65-year-olds revealed higher glycolytic glucose uptake in elderly B cells, with aging stimulating increased glucose uptake [93]. Meanwhile, Cerebrospinal fluid (CSF) lactate measurements in 42 men and 102 women (16-69 years) showed adult CSF lactate trended upward after age 54, with no sex-based difference [94].Autopsies on the cerebral cortex of elderly AD patients revealed an increase in Tau lactylation of K331 in the brain [10].

While lactate levels rise with aging, accumulation primarily occurs in brain and muscle. Whether liver, heart, and other tissues exhibit similar age-related lactate elevation remains unclear. Quantitative analysis of age-related lactate dynamics across different age groups, coupled with non-invasive lactate-sensitive magnetic resonance spectroscopy (MRS), could advance aging research and facilitate anti-aging drug development (Table 2).

## 6. The effect of lactylation modification on the mechanism of aging

Lactate levels increase with age, leading to the occurrence of lactylation. The occurrence of lactylation, in turn, aggravates the aging and functional decline of the body through multiple pathways. Some studies have taken this as a basis for treating lactylation modification as a target to treat and prevent aging-related diseases (Table 3).

### 6.1 Cellular senescence

Cellular senescence, a cardinal feature of aging, is characterized by irreversible cell cycle arrest triggered by injury or stress. Senescent cells secrete a pathogenic senescence-associated secretory phenotype (SASP), which promotes secondary senescence, disrupts tissue homeostasis, and compromises repair mechanisms [107].

Elevated lactate exacerbates cell senescence by promoting SASP release. In vascular smooth muscle cells (VSMCs), aging-induced TRAP1 upregulation enhances aerobic glycolysis, driving lactate accumulation. Lactate promotes histone H4K12 lactylation, which enriches SASP gene promoters to activate transcription and intensify senescence. Studies show TRAP1 mediates metabolic reprogramming via HDAC3, enhancing lactate-dependent H4K12la to promote SASP expression [103]. In the brain, elevated lactate promotes H3K18 lactylation in senescent microglia and hippocampus of naturally aging mice, directly activating NFκB signaling to enhance SASP release and accelerate aging [100]. lactylation may regulate aging by modulating specific protein activity. Studies show LKB1 induces lactate production, inhibiting histone H4 Lys8 and Lys16 lactylation to alter Sp1-related transcriptional activity. Sp1 mediates TERT (telomerase reverse transcriptase) inhibition following LKB1 overexpression, inducing cellular senescence [98].

**Table 3:** Potential lactylation targets involved in aging-related diseases.

Target Type	Target Name	Regulatory Direction	Related Aging Phenotype	Associated Aging Disease	Potential Intervention Evidence	Ref.
**Delactylase**	HDAC2	Inhibition	Retinal microvascular endothelial cell senescence	Age-related macular degeneration	Under VEGF stimulation, LDHA expression increases in HRMECs, lactate levels rise, upregulation of H3K9 lactylation suppresses HDAC2 expression. HDAC2 overexpression reduces H3K9 lactylation and inhibits angiogenesis	[59]
	HDAC3	Inhibition	Reduced collagen synthesis in fibroblasts	Skin aging	HDAC3 inhibitor disrupts lactate-H4K12la-TGF-β axis in macrophages, inhibiting collagen synthesis in fibroblasts	[97]
**Lactyltransferase**	P300	Promotion	Tau protein metabolism	AD	tau lactylation occurs with p300 and lactoyl-CoA; no lactylation detected with acetyl-CoA	[10]
**Metabolic Enzyme**	PKM2	Promotion	Reduced osteoblast differentiation	Osteoporosis	EC-specific Pkm2 deletion lowers serum lactate, downregulates histone lactylation, limiting osteoblast differentiation	[30]
	AMPK	Promotion	Decline in autophagy	Intervertebral disc degeneration	Glutamine inhibits glycolysis, reduces lactate production, downregulates AMPK lactylation, suppresses NP cell senescence	[95]
**Transcription Factor/Cofactor**	SP1	Inhibition	Lung adenocarcinoma cell senescence	Lung cancer	Downregulation of H3/H4 lactylation reduces Sp1 activity, blocks TERT transcription, inhibits senescence and telomerase activity	[98]
	Glis1	Downregulation	Renal tubular cell senescence	Age-related renal fibrosis	Glis1 overexpression reduces histone-KAT5 binding affinity, potentially lowering lactylation and suppressing renal tubular cell senescence	[99]
	NFκB	Promotion	Microglial senescence	AD	Increased brain lactate → H3K18 lactylation → stimulates NFκB signaling → promotes SASP release	[100]
	FOXO1	Promotion	Increased mitochondrial oxidative stress	<mark>Age-related diabetes</mark>	Increased H4K12la activates FOXO1/PGC-1α pathway → promotes mitochondrial oxidative stress	[101]
**Gene**	Lactylation-related gene CBX3	Promotes histone lactylation	NP cell senescence	Intervertebral disc degeneration	Atosiban acetate targets CBX3, inhibits glycolysis, downregulates lactate production and histone lactylation, promotes matrix synthesis	[102]
**Receptor**	TRAP1	Promotion	Vascular smooth muscle cell senescence	Atherosclerosis	TRAP1 knockout inhibits glycolysis, reduces lactate, increases HDAC3 expression, downregulates H4K12la, inhibits SASP transcription/secretion	[103]
**Non-coding RNA**	EPB41L4A-AS1	Promotion	Decline in autophagy	AD	EPB41L4A-AS1 knockdown reduces GCN5L2 expression, downregulates histone lactylation near autophagy genes, increases autophagy gene transcription, mediates Aβ clearance	[104]
**Specific Protein Site**	Tau K677	Inhibition	Decline in autophagy	AD	K677R mutation downregulates tau lactylation; lactylated tau K677 mediates MAPK pathway, inhibits ferritinophagy, reduces ferroptosis	[105]
	Amyloid precursor protein K612 (APP K612)	Promotion	Increased Aβ clearance via lysosomal degradation	AD	Lactylated APP inhibits BACE1 binding, promotes APP-CD2AP interaction, facilitates APP degradation via endosome-lysosome pathway	[106]

Lactate-mediated regulation of cell senescence is evident in aging-related diseases. GLIS1 (GLIS family zinc finger 1) attenuates age-related renal fibrosis: overexpression reduces renal tubular epithelial cell (RTEC) senescence in DKD mice by downregulating histone lactylation. lactylation enhancers diminish GLIS1’s protective effect against cell senescence, compromising its anti-aging capacity [99]. lactylation inhibitor enhances Glis1 activity. Additionally, Glis1 reprograms senescent cells into pluripotent cells, improves genomic stability, and alleviates cellular senescence [54].

While studies have shown lactylation drives cellular senescence—particularly SASP release—substantial research gaps and unexplored directions remain. Systematic comparative studies are needed to clarify its regulatory networks and key targets. Current research primarily focuses on histone lactylation, leaving lactylation of non-histone proteins (e.g., signaling pathway key proteins, transcription factors, metabolic enzymes) and their roles in aging regulation largely uninvestigated—a vast frontier in this field.

### 6.2 Mitochondrial function

Mitochondrial dysfunction is a significant indicator of aging and can also accelerate the aging process. This acceleration is specifically manifested as excessive production of reactive oxygen species (ROS) within mitochondria, oxidative damage to mitochondrial components, reduced ATP supply capacity, tricarboxylic acid cycle disorders, and increased mitochondrial-driven inflammation [108]. It is notable that L-lactate, as the end product of glycolysis and the substrate of mitochondrial respiration, plays a pivotal role in connecting anaerobic and aerobic metabolism [109]. Under physiological conditions, pyruvate produced by glucose metabolism is mainly consumed by the oxidative phosphorylation (OXPHOS) pathway in mitochondria to generate ATP. However, under hypoxic conditions, mitochondrial protein lactoylation inhibits OXPHOS through a dual mechanism: On the one hand, by inducing the abnormal accumulation of alanine-trNA synthase (AARS2), and on the other hand, through the lactylation modification at the PDHA1 lysine 336 and CPT2 lysine 457/8 sites, it blocks the influx of acetyl-CoA produced by the oxidation of pyruvate and fatty acids, ultimately leading to the inactivation of key enzymes and the inhibition of ATP production [110].

Recent studies have found that protein lactylation modification has a regulatory effect on aging-related mitochondrial dysfunction. The lactylation of specific mitochondrial proteins can directly affect the electron transport efficiency, ATP synthesis ability and mitochondrial membrane integrity. Animal experiments have shown that the abnormal activity of mitochondrial respiratory chain enzymes in the brain regions of premature aging model mice is closely related to the changes in the transcriptional activity of lactate dehydrogenase. This change significantly increases the level of cerebral lactate by promoting the conversion of pyruvate to lactate [88]. Further in-depth research has revealed that the lactylation modification of the ALDH2 protein can trigger the ubiquitination degradation of PHB2, thereby inhibiting mitochondrial autophagy and exacerbating functional disorders [111]. This obstacle to the clearance of damaged mitochondria may form a vicious cycle and accelerate the process of cellular aging.

At the disease level, the association between lactylation modification and age-related diseases has been experimentally supported. In the brains of mice with diabetes mellitus (T2DM) models, microglia show lactate accumulation. By inducing the upregulation of histone lactoylation (Kla) at the H4K12 site, the FOXO1/PGC-1α signaling pathway is activated and mitochondrial oxidative stress is triggered [101]. Furthermore, the mitochondrial fragmentation phenotype has been found to mediate epigenetic reprogramming by driving histone lactoylation, thereby regulating the decomposition response of macrophages in inflammatory injury [112].

While studies have shown lactylation contributes to age-related mitochondrial dysfunction, such as inducing AARS2 misaccumulation and inhibiting OXPHOS via PDHA1 Lys336 and CPT2 Lys457/8 lactylation, key knowledge gaps remain. lactylation sites in mitochondrial respiratory chain complexes (CI-CV) and transporters (e.g., VDAC) remain uncharacterized. Age-dependent modification dynamics and their impact on electron transport efficiency are unknown, and the interplay between lactylation networks and mitochondrial homeostasis requires in-depth exploration.

### 6.3 Autophagy

Autophagy is a conserved lysosomal degradation process. It promotes cellular homeostasis and stress adaptation by phagocytosing cytoplasmic components (including damaged organelles) in double-membrane organelles (autophagosomes) and then transporting these components to lysosomes for degradation [113, 114]. The occurrence of lactylation can directly act on autophagy-related proteins to a certain extent and promote the occurrence of autophagy. In rat cardiac cells, H3K18R mutation treatment led to a significant decrease in the level of mitochondrial autophagy-related protein LC3II, inhibiting the occurrence of mitochondrial autophagy [115]. The lactylation of aldehyde dehydrogenase 2 (ALDH2) can promote the ubiquitination - proteasome degradation of mitochondrial autophagy receptor (PHB2), thereby inhibiting mitochondrial autophagy, directly affecting mitochondrial function and aggravating mitochondrial dysfunction [111].

IIn addition to directly acting on autophagy-related proteins, lactylation modification also indirectly affects the occurrence of autophagy by mediating the activities of related enzymes. Specifically, the lactoylation modification of PIK3C3/VPS34 can enhance its binding ability with autophagy-related factors. By increasing the lipid kinase activity of PIK3C3/VPS34, it simultaneously promotes the efficiency of the autophagy process and the endolysosomal degradation pathway [116, 117]. Moreover, lactate molecules can covalently conjugate to the transcription factor TFEB, inducing its lactoacylation and sustaining structural stability. Lactoacylated TFEB preferentially interacts with the E3 ubiquitin ligase WWP2, inhibiting its own ubiquitination and proteasomal degradation pathways, thereby culminating in enhanced TFEB activity and augmented autophagic flux [77].

In the process of aging and aging-related diseases, lactylation modification plays a key role. It can influence the development trajectory of aging by improving the autophagy process. Take intervertebral disc degeneration (IVDD) as an example. As a common age-related degenerative disease, studies have clearly pointed out that the level of lacylation is positively correlated with IVDD, which means that the higher the level of lacylation, the more severe the degree of intervertebral disc degeneration may be. Based on this, the characteristics of lactylation-related genes (LRG) are expected to become biomarkers for the effective clinical treatment of IVDD [102]. Furthermore, glutamine also plays an important role in it. It can reduce the lactylation of AMPKα, thereby affecting the autophagy process and ultimately achieving the effect of inhibiting the senescence of N cells in the nucleus pulposus [95]. In Alzheimer's disease (AD), lactylation modulates pathology via dual mechanisms. The long non-coding RNA EPB41L4A-AS1 is downregulated in AD patients, altering histone modifications at autophagy gene promoters by regulating GCN2 expression. This inhibits glial cell-mediated Aβ clearance [104].On the other hand, Tau protein K677 lactylation regulates iron metabolism homeostasis in AD model mice. Mutations at this site (K677R) reduce tau lactylation, inhibiting ferroptosis by upregulating NCOA4, FTH1, and other factors. This involves MAPK-mediated ferritin autophagy regulation, offering new therapeutic strategies for AD [105].

However, the research on the regulation of autophagy by lactylation is highly dependent on indirect evidence, and there is a lack of direct visualization tools to prove that lactylation modification dynamically regulates autophagosome formation. Visualization analysis can be conducted by developing LC3fluorescent probes modified by lactylation. In addition, the existing evidence mainly comes from specific cell models or specific diseases. The lactate-autophagy axis is present in various tissues, cell types, and broader aging-related diseases. The universal and specific mechanisms in cardiovascular aging, osteoarthritis, and metabolic diseases remain unknown. Exploring the synergistic effect of targeted lactylation in combination with existing autophagy regulators or disease treatment strategies holds significant translational prospects.

**Figure 2.**
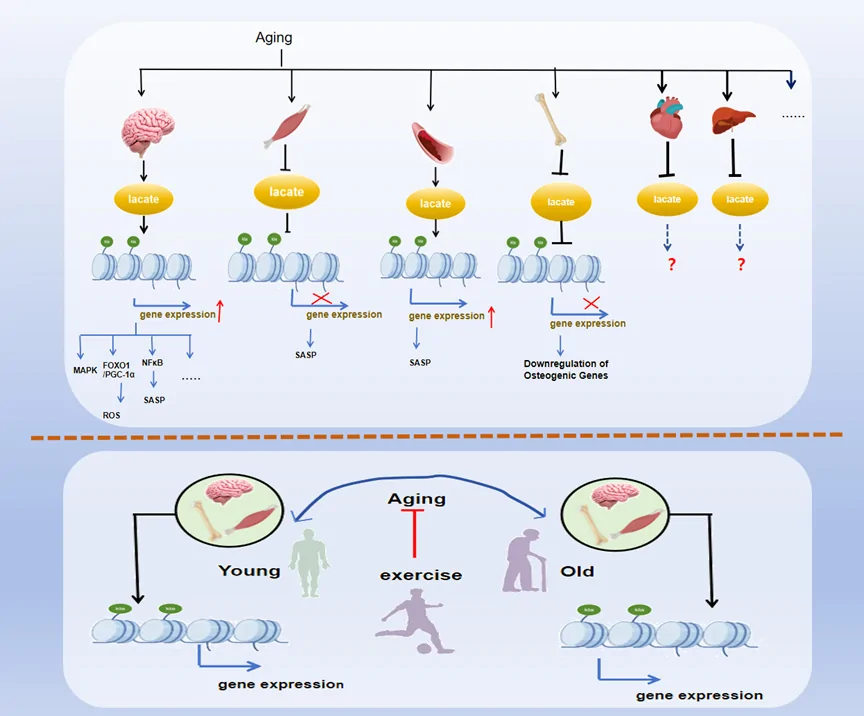
**During the aging process, the changes in lactate content and lactylation levels vary among different tissues**. They show an upward trend in the brain and vascular smooth muscle, while the opposite trend occurs in skeletal muscle and bone. Meanwhile, only some literature has suggested that the lactate levels in the heart and liver decline during aging, but the systemic changes in lactylation levels have not been deeply studied. The existing research on the mechanism of lactate regulation of aging has focused more on brain tissue, with relatively less exploration in other tissues. Exogenous lactate produced by exercise has an anti-aging effect on the brain, skeletal muscles and bones.

## 7. Existing disputes, technical challenges and future directions

### 7.1 Lactate modification is the current technical defect

Existing evidence in humans and mice shows a correlation between lactate accumulation and lactylation levels during aging [88, 92, 93]. Gaps exist in the "lactate-lactylation-aging" causal chain, compounded by current detection technologies' limited spatial resolution. This impedes single-cell analysis of lactylation heterogeneity, despite aging being a highly heterogeneous process. Single-cell lactylation heterogeneity analysis remains challenging. While the FiLa lactate probe enables dynamic single-cell lactate monitoring, it has not been directly integrated with lactylation detection [118]. Aging is inherently heterogeneous, necessitating the development of more targeted detection technologies to advance this field.

Quantitative analysis remains challenging for accurately measuring lactylation dynamics. Aging's gradual nature implies lactylation may exhibit nonlinear age-dependent changes, with distinct metabolic and modificatory dynamics across senescence stages. However, Current aging induction methods typically simulate only terminal aging, comparing "young" and "old" time points, thus failing to capture intermediate dynamic details or accurately track lactylation dynamics across senescence stages [30, 119]. The lactylation dynamic technology that can simulate human tissue aging using organoids can monitor the dynamic changes of lactylation during aging.

Studying lactylation's role in aging requires a targeted toolset for writing, erasing, and reading these modifications. While p300/CBP acetyltransferases and HDAC deacetylases are implicated in lactylation regulation, these enzymes act on both lacto- and acetylation modifications simultaneously [73]. Determinants of lysine acetylation vs. lactylation remain unknown. The absence of highly specific lactylation writers/erasers hinders precise manipulation of protein lactylation states for functional studies. Future breakthroughs may lie in developing site-specific nano-single-domain antibodies against histone lactylation and constructing inducible tissue-specific lactate enzyme knockout models.

### 7.2 Research differences with other mechanisms of aging

Existing studies have established lactylation’s driving role in aging via molecular mechanistic analyses and animal models. Notably, significant gaps remain in understanding its upstream enzyme regulatory networks and interactions with other aging mechanisms (oxidative stress, inflammation, telomere shortening—among others). Substantial knowledge deficits also exist regarding lactylation’s relationship with circadian rhythms and the intestinal microbiota in aging. Emerging evidence hints at links between lactylation and circadian regulation, identifying this as a promising research node for future inquiry.

**Figure 3.**
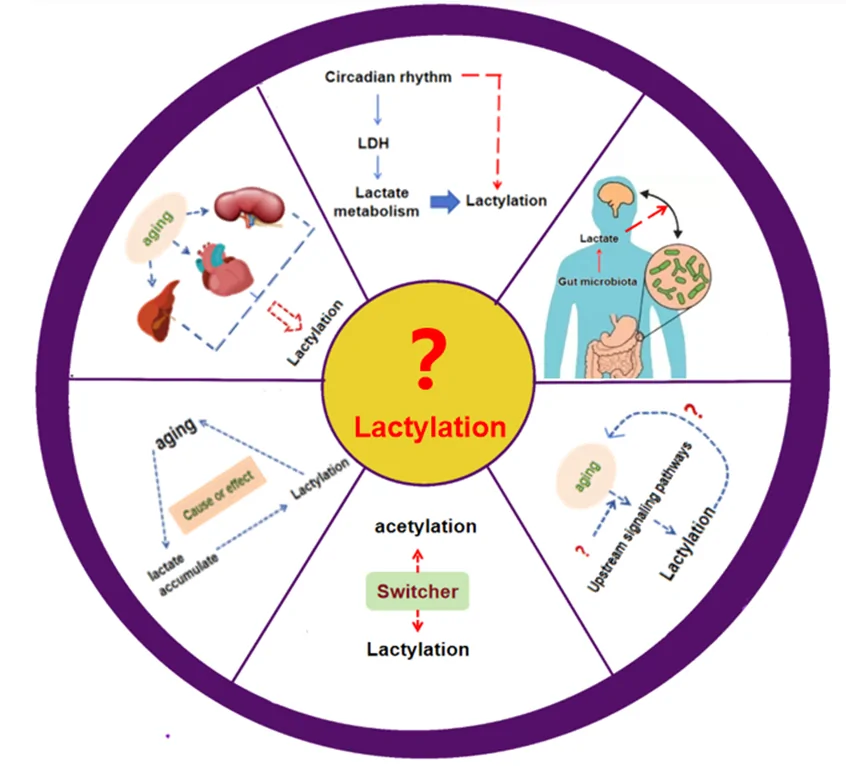
**Problems to be solved in the field of aging through lactylation modification**. (1) The causal relationship chain of aging - lactate accumulation - lactylation modification; (2 ) The specific pathways by which the upstream pathways of lactylation play a role in aging; (3) The determining factors that decide whether lysine undergoes acetylation or lactylation; (4) Research data on lactylationin other aging tissues; (5) The circadian rhythm can regulate the activity of LDH and affect the generation and clearance of lactate, and whether it directly mediates the occurrence of lactylation; (6) Intestinal microbiota can produce lactate. Does it affect the lactylation modification in the brain through the gut-brain axis and influence the aging process.

Disruption of circadian rhythm can accelerate the aging process through several aspects. Glucose, fatty acid, and cholesterol metabolic pathways are controlled by circadian rhythms, and disruption of clock genes can alter metabolism and worsen health [120]. An experiment to assess brain lactate levels in young and elderly healthy female volunteers during 40 hours of sleep deprivation. Lactate levels can rise by up to 40% during brain function tasks in young subjects, but aging or sleep deprivation can of brain energy metabolism [123]. In animal studies, lactate levels in the prefrontal cortex of young and old mice were higher at night than during the day, when the mice were in a light-dark cycle. However, lactate levels increased earlier in the aged mice than in the young mice [121]. The transcriptional level of lactate dehydrogenase (LDH) is closely related to the increase in lactate concentration and circulates in the head of elderly fruit flies at a circadian rhythm [122]. This suggests that circadian clock genes may affect lactate production and clearance by regulating LDH activity. Based on the above studies, we hypothesized that circadian rhythm may affect the occurrence of lactate production. Future studies can combine time-resolved metabolomics and epigenomics to map the circadian oscillations of lactylation during aging, focusing on whether core clock proteins (such as PER2) are modified by lactylation and the regulation mechanism of rhythm on lactatase activity.

Intestinal flora imbalance has been listed as one of the new markers of aging. lactate is both a host metabolite and a metabolic by-product of intestinal microbiota. Excessive production can lead to the lactylation of histones [123]. Is altered metabolism of the intestinal microbiota during aging a potential source of lactate accumulation? At the same time, the gut interacts with the brain in a bidirectional manner, called gut-brain axis. This interaction is carried out through three main pathways, namely immune pathway, neuronal pathway and endocrine/systemic pathway [124]. Based on the above, we speculate that in addition to lactate produced by the brain itself, lactate produced by gut microbes can affect the modification of lactate in the brain through the gut-brain axis and affect the occurrence of aging. To answer these questions, it is necessary to combine microbiome and metabolic flow analysis to clarify the contribution of microbial metabolism to host lactylation modification.

While brain and muscle lactylation are well-studied in aging, data remain scarce for metabolic organs like liver and heart. Tissue-specific mitochondrial oxidation/glycolysis differences may drive lactylation functional divergence in aging—aging increases brain lactylation, while muscle shows the opposite trend—highlighting the need to analyze tissue microenvironment effects on lactylation. Upstream regulatory pathways like Hippo may also modulate lactylation, as seen in other diseases [125, 126]. But it has not yet been reflected in the aging mechanism; Through in-depth research on the above, it is helpful to reveal the complete mechanism pathway of lactylation in aging (Fig. 3).

## 8. Conclusions and Prospects

As an emerging protein post-translational modification, lactylation is intricately linked to aging. During senescence, elevated intracellular lactate triggers widespread lactylation events, modulating cellular functions via multiple pathways to drive aging-related diseases. Current lactylation research remains in its infancy, with pronounced gaps. As aging intensifies, the need to expand anti-aging strategies and deepen mechanistic research grows. Deciphering lactate-aging interconnections will illuminate tissue-specific aging mechanisms, providing precise theoretical foundations for treating tissue-specific aging diseases. This will also unravel the global molecular regulation of aging, offering novel targets for comprehensive anti-aging strategies.
